# Effect of a Rehabilitation Program After Mesenchymal Stromal Cell Transplantation for Advanced Osteonecrosis of the Femoral Head: A 10-Year Follow-Up Study

**DOI:** 10.1016/j.arrct.2022.100179

**Published:** 2022-01-13

**Authors:** Tomoki Aoyama, Koji Goto, Ryosuke Ikeguchi, Manabu Nankaku, Katsuyuki Madoba, Momoko Nagai-Tanima, Akira Ito, Ryosuke Kakinoki, Takashi Nakamura, Shuichi Matsuda, Junya Toguchida

**Affiliations:** a Department of Physical Therapy, Human Health Sciences, Graduate School of Medicine, Kyoto University, Kyoto, Japan; b Department of Rehabilitation, Kyoto University Hospital, Kyoto, Japan; c Department of Orthopedic Surgery, Graduate School of Medicine, Kyoto University, Kyoto, Japan; d Rehabilitation Unit, Kyoto University Hospital, Kyoto, Japan; e Department of Rehabilitation, Kyoto Hakuaikai Hospital, Kyoto, Japan; f Department of Tissue Regeneration, Institute for Frontier Medical Sciences, Kyoto University, Kyoto, Japan; g Center for iPS Cell Research and Application, Kyoto University, Kyoto, Japan

**Keywords:** Femur head, Mesenchymal stem cells, Osteonecrosis, Regenerative medicine, Rehabilitation

## Abstract

**Objective:**

To assess the status of 10 patients with advanced osteonecrosis of the femoral head who underwent mesenchymal stromal cell transplants and a 12-week rehabilitation program 10 years earlier.

**Design:**

Retrospective study.

**Setting:**

University clinical research laboratory.

**Participants:**

Patients (N=10) who had undergone mesenchymal stromal cell transplantation and rehabilitation for a single hip osteonecrosis of the femoral head 10 years prior to the current study were recruited by telephone. The average age was 31.7 years and all participants were men; radiographic stages were 3A in 6 patients and 3B in 4 patients before treatment.

**Intervention:**

A 12-week rehabilitation program with follow-up once every 1 to 2 years was performed after mesenchymal stromal cell transplantation.

**Main Outcome Measures:**

Radiographic analysis, clinical score, timed Up and Go test, hip function (range of motion, muscle strength), and Short Form-36 scores were assessed before treatment and 1 and 10 years after treatment.

**Results:**

Upon imaging, 5 hips were found to be stable (stable group) and 5 had progressed (progressed group); 2 of the latter group required a total hip arthroplasty. The pretreatment radiographic stage of the progressed group was more advanced than that of the stable group. Body mass index was higher in the progressed group than in the stable group. Hip function and clinical score at 1 and 10 years after treatment improved in the hips of 8 patients without total hip arthroplasty. There were no severe adverse events during the rehabilitation.

**Conclusions:**

The 12-week rehabilitation program and annual follow-up after mesenchymal stromal cell transplantation for osteonecrosis of the femoral head was associated with pain reduction, maintaining hip muscle strength, widening range of motion, and improving quality of life. The level and timing of weight-bearing and social activity should be planned according to the individual's lifestyle and body composition.

Osteonecrosis of the femoral head is a painful disorder that progresses to femoral head collapse and osteoarthritis of the hip joint.^1^^,^^2^ Osteonecrosis of the femoral head mainly affects younger adults and accounts for 10% of total hip arthroplasties in the Unites States annually.^2^ Nonoperative treatment modalities are limited only to cases in which the necrosis is small and medially located.^2^ When the disorder progresses, the patient requires total hip arthroplasty.^1^, ^2^, ^3^

Although total hip arthroplasty is superior, joint-preserving treatment is preferred for younger patients. However, joint-preserving procedures should attempt to save the femoral head.^2^ Recently, cell-based procedures have been increasingly reported as a joint-preserving modality. Mesenchymal stromal cell transplantation in combination with core decompression surgery has been performed since the early 21st century and has been found to significantly delay femoral head collapse during the precollapse stage.^4^, ^5^, ^6^, ^7^, ^8^, ^9^ However, in more advanced stages, the results of this procedure have been unsatisfactory.^2^ In response, Aoyama et al designed a protocol using a combination of cultured mesenchymal stromal cells and vascularized bone grafts for advanced stages of osteonecrosis of the femoral head.^10^ As a result, their 2- and 10-year radiographic and clinical results indicated that the protocol is effective.^10^^,^^11^

Nevertheless, except for one report evaluating rehabilitation after cell transplantation for osteonecrosis of the femoral head,^12^ there have been few reports on this subject. Moreover, discussion on the effect of rehabilitation alone for osteonecrosis of the femoral head is insufficient.^13^^,^^14^ However, recent research has shown that there is a synergistic effect when rehabilitation is combined with cell transplantation.^15^, ^16^, ^17^, ^18^ The findings of Aoyama et al suggest that a rehabilitation program is feasible after cell transplantation,^12^ and their 10-year radiographic and clinical results demonstrate that cell transplantation can be useful.^11^ The current study aims to evaluate the usefulness of the rehabilitation programme after mesenchymal stromal cell transplantation for osteonecrosis of the femoral head through physical and functional assessments 10 years after treatment.

## Methods

The original study was a prospective case series of patients enrolled in a clinical trial conducted at a university hospital in Japan.^10^ The original study (C83) and the current 10-year follow-up study (R1950) were approved by the hospital ethics committee and were conducted according to the principles of the Declaration of Helsinki. The original clinical trial was registered in the University Hospital Medical Institution Network Clinical Trial Registry, and 10 patients participated. For the current study, participants were recruited by telephone. The current study was performed as a secondary analysis of the original 10-year follow-up study that evaluated the radiographic and clinical results of mesenchymal stromal cell transplantation for osteonecrosis of the femoral head.^11^

### Radiographic assessment

The staging of osteonecrosis of the femoral head proposed by the Japan Investigation Committee is a modified version of the system proposed by the Association Research Circulation Osseous committee.^19^ Necrotic lesion type and size were assessed according to the radiographic classification proposed by the Japan Investigation Committee.^19^

### Inclusion criteria

Patients with a single hip at radiographic stage 3A or 3B according to the Japan Investigation Committee staging^19^ and who were aged 20 to 50 years old were included in the original study. For the current study, we included patients 10 years after they had undergone mesenchymal stromal cell transplantation for osteonecrosis of the femoral head and rehabilitation in the original clinical trial. Written informed consent was obtained from all the participants in the clinical study.

### Mesenchymal stromal cell transplantation augmented by vascularized bone grafting

The necrotic area was removed by curettage under both fluoroscopic and endoscopic guidance. Mesenchymal stromal cells (0.5-1.5 × 10^8^) premixed with β-tricalcium phosphate granules^a^ were transplanted into the created cavity. The tricortical iliac crest bone with a vascular pedicle was grafted into the bone.^10^

### Rehabilitation program

Patients were hospitalized and rehabilitation was performed for 12 weeks after surgery. Weight bearing was not permitted for the first 6 weeks after transplantation surgery; subsequently, one-third weight bearing, one-half weight bearing, and two-thirds weight bearing were allowed, progressing at 2-week intervals for each. Full weight-bearing was permitted 12 weeks after the treatment. Details of the rehabilitation program, including range of motion exercises, muscle strengthening exercises, and aerobic training, were reported previously.^12^ The entire rehabilitation program was supervised by skilled physiotherapists, and the specific therapy received was recorded in the participants’ medical records. After the 12-week rehabilitation program, follow-up and lifestyle checks, such as weight control, smoking cessation, and participation in social activity, were performed once every 1 to 2 years.

### Evaluations

Measurements were performed at pretreatment and 1 and 10 years after treatment. Body mass index (kg/m^2^) was calculated by measuring the height and weight of each participant. Progression of osteonecrosis of the femoral head was measured according to the radiographic stage established by the Japan Investigation Committee.^19^ Clinical outcome was evaluated using the Japanese Orthopedic Association score.^20^ For hip functional assessment, passive hip flexion, extension, abduction, and external rotation angles were measured using universal goniometry. Hip flexor, extensor, and abductor strengths were measured using a handheld dynamometer^b^ during isometric contraction for 3 seconds with manual resistance. Knee extensor and flexor strengths were assessed using the Iso Force GT-330.^c^ Torque was expressed as a percentage of body weight (Nm/kg). For the timed Up and Go test, the time to stand from an armless chair, walk a distance of 3 meters, turn, walk back to the chair, and sit down was measured. Health-related quality of life was evaluated using the Short Form-36.^21^ The Short Form-36 was categorized into physical functioning, role limitations due to physical functioning, bodily pain, general health, social functioning, and role functional subgroup scores.

### Adverse events

In the original study, adverse events were monitored by the Department of Clinical Trial Design and Management Translational Research Center. After the original clinical trial, adverse events were monitored at the follow-up.

### Statistical analysis

Body mass index, Brinkman index, timed Up and Go test, range of motion, muscle strength, and Short Form-36 score were described as the mean ± SE. Independent *t* tests were performed for age, Brinkman index, and body mass index. Stepwise logistic regression analysis was performed for the Japanese Orthopedic Association score, range of motion, and muscle strength. All statistical analyses were carried out using JMP IN (version 15).^d^ Statistical significance was set at a *P* value less than .05.

## Results

### Demographic analysis

Ten patients participated in the original clinical trial conducted from November 2007 to June 2009. All 10 patients participated in the 10-year follow-up study between March 2019 and March 2021 (table 1). Five of the hips (patients 2, 4, 5, 9, and 10) remained stable without progression to osteoarthritis (stable group), and 5 hips (patients 1, 3, 6, 7, and 8) progressed to the osteoarthritic stage (progressed group). The pretreatment radiographic stage in all stable-group hips was 3A (table 1). One stage 3A hip (patient 6) and 4 stage 3B hips (patients 1, 8, 3, and 7) progressed to stage 4 at the 10-year follow-up. Among the progressed-group hips, 2 (patients 3 and 7) underwent total hip arthroplasty 6 and 8 years after transplantation (supplemental fig S1, available online only at http://www.archives-pmr.org/).

**Table 1:** Patient data

	Baseline Data							Bodily Composition										Radiographic Evaluation						
								Height, cm	Weight, kg			BMI, kg/m^2^			Thigh Circumference, cm			Type*	JIC Stage: Tx Side†			JIC Stage: Non-Tx Side†		
Group	Patient No.	Age, years	Sex	TS	History	Brinkman index	SU		Pre-Tx	1Y	10Y	Pre-Tx	1Y	10Y	Pre-Tx	1Y	10Y		Pre-Tx	1Y	10Y	Pre-Tx	1Y	10Y
Stable group	2	23	M	L	Cushing syndrome	0	Y	171.0	56.6	57.6	60.2	19.4	19.7	20.6	39.0	38.5	41.0	C2	3A	3A	3A	2	2	2
	4	20	M	R	Hepatitis	0	Y	174.2	76.8	72.9	70.6	25.3	24.0	23.3	50.5	50.0	53.5	C1	3A	3A	3A	2	2	2
	5	35	M	L	None	300	N	178.8	70.0	59.2	62.0	21.9	18.5	19.4	43.2	38.5	38.0	C2	3A	3A	3A	THA	THA	THA
	9	33	M	R	None	0	N	174.2	61.0	65.7	70.0	20.1	21.7	23.1	42.5	42.5	44.0	C2	3A	3A	3A	1	1	1
	10	38	M	R	None	300	N	166.7	52.9	52.5	54.4	19.0	18.9	19.6	40.0	38.7	38.5	C2	3A	3A	3A	3A	3B	THA
Progressed group	1	27	M	R	Nephritis	300	Y	170.9	66.5	68.7	62.0	22.8	23.5	21.2	42.5	42.0	41.0	C2	3B	3B	4	2	2	2
	6	28	M	R	None	300	N	169.2	58.3	63.9	53.4	20.4	22.3	18.7	40.0	44.2	42.5	C2	3A	3A	4	2	2	2
	8	26	M	R	None	105	N	175.1	66.4	73.4	65.0	21.7	23.9	21.2	44.0	47.2	46.5	C2	3B	3B	4	4	4	4
	3	48	M	R	Meningioma	72	N	174.7	87.5	69.6	84.0	28.7	22.8	27.5	46.2	40.0	41.0	C2	3B	3B	THA	2	2	2
	7	39	M	R	Leukemia	0	Y	183.1	85.2	85.4	80.9	25.4	25.5	24.1	50.2	49.8	47.0	C2	3B	3B	THA	2	3A	THA

*Abbreviations:* BMI, body mass index; L, left; M, male; N, no; R, right; SU, steroid use; THA, total hip arthroplasty; TS, treatment side; Tx, treatment; Y, yes.

⁎ Radiogenic classification Japanese Investigation Committee.

† Modified version of the radiographic staging system proposed by the American Research Circulation Osseous Committee.

To analyze the cause of the collapse of the femoral head, the demographic data of the stable and progressed groups were compared. The age of patients at the time of treatment was greater in the progressed group (33.6±4.3y) than in the stable group (29.8±3.5y); however, the difference was not significant. All participants had succeeded in stopping smoking when they participated in the clinical trial and were still not smoking at the time of the 10-year follow-up. The Brinkman index at the time of treatment was lower in the stable group (120±73.5) than in the progressed group (155.4±61.4); however, the difference was not significant (*P*=.7). Body mass index at pretreatment was higher in the progressed group (23.8±1.4 kg/m^2^) than in the stable group (21.1±1.2 kg/m^2^); however, the difference was not significant (*P*=.5). Body mass index at 1 year after the treatment was higher in the progressed group (23.7±0.6 kg/m^2^) than in the stable group (20.6±1.0 kg/m^2^); this difference was significant (*P*<.05). Body mass index at 10 years after the treatment was not different between the stable (21.2±0.8 kg/m^2^) and progressed (22.9±0.4 kg/m^2^) groups. The type of necrotic area was not different between the stable and progressed groups (table 1).

### Clinical results

**Fig 1:**
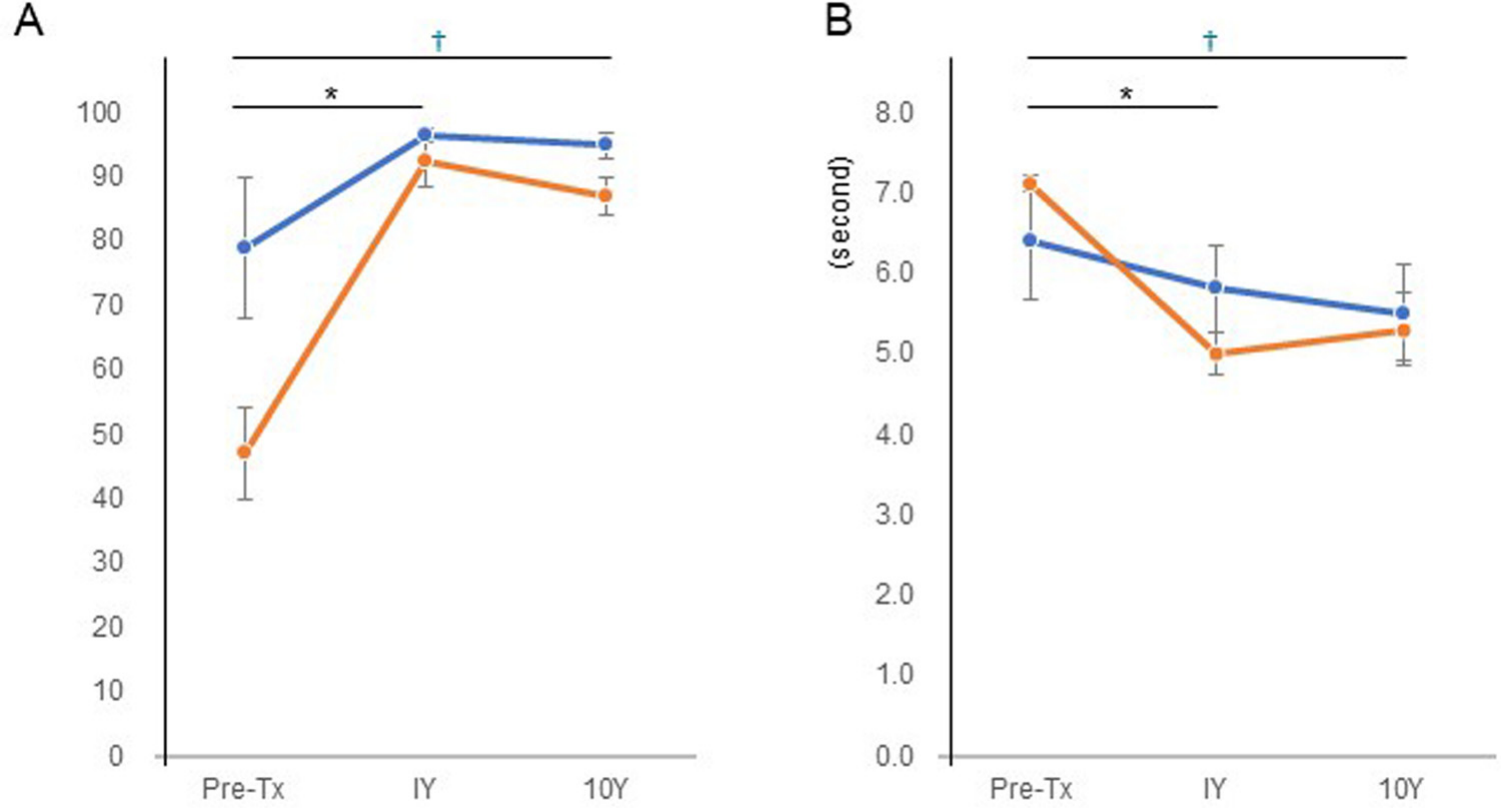
Transition of clinical results. (A) Japanese Orthopaedic Association score for hips. (B) timed Up and Go test (in seconds). The blue line indicates the average of the stable group (n=5); the orange line indicates the average of the progressed group without total hip arthroplasty (n=3). *Significant difference between pretreatment and 1 year after the treatment. ^†^Significant difference between pretreatment and 10 years after treatment. *P*<.05. Tx, treatment; Y, year.

The clinical results of total hip arthroplasty were determined, and the transitions of the clinical and physiological results were compared between the 5 stable hips (patients 2, 4, 5, 9, and 10) and the 3 progressed hips without total hip arthroplasty (patients 1, 6, and 8). The clinical score (Japanese Orthopedic Association score) improved in both the stable group and the progressed group without total hip arthroplasty (fig 1A); however, the score was not significantly different between the 2 groups. The timed Up and Go test was significantly improved in both the stable group and the progressed group without total hip arthroplasty (fig 1B); however, the time taken to complete the test was not significantly different between the 2 groups.

### Hip range of motion

**Fig 2:**
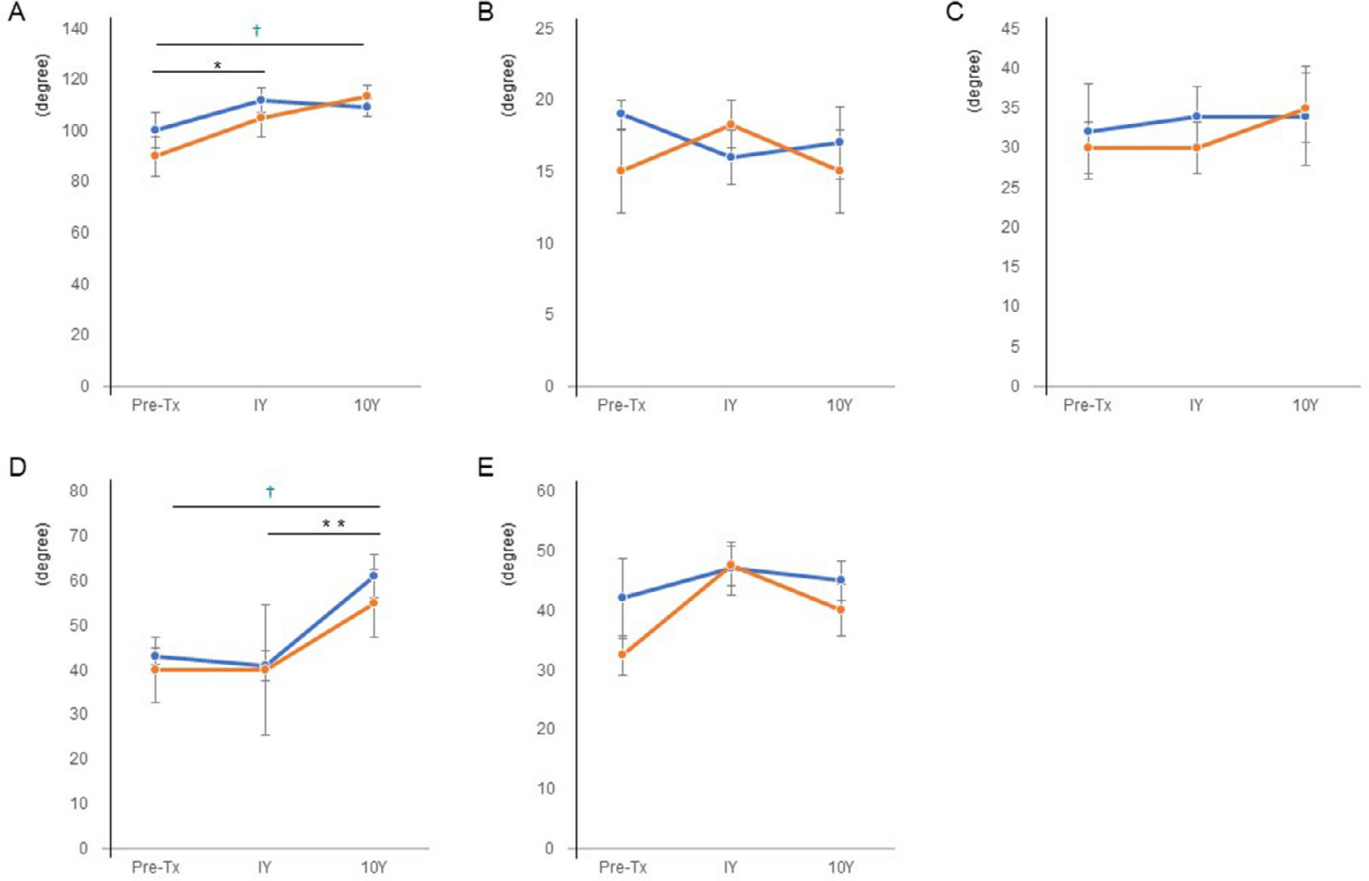
Transition of hip range of motion: (A) hip flexion, (B) hip extension, (C) hip abduction, (D) straight leg riding, and (E) hip outer rotation (in degrees). The blue line indicates the average of the stable group (n=5); the orange line indicates the average of the progressed group without total hip arthroplasty (n=3). *Significant difference between pretreatment and 1 year after treatment. **Significant difference between 1 year and 10 years after treatment. ^†^Significant difference between pretreatment and 10 years after treatment. *P*<.05. Tx, treatment; Y, year.

In both the stable group and the progressed group without total hip arthroplasty, the hip range of motion improved at 1 and 10 years after treatment (fig 2). There was a significant improvement in flexion (fig 2A) and straight leg riding (fig 2D) in both the stable group and the progressed group without total hip arthroplasty; however, there was no significant difference between the groups.

### Muscle strength

**Fig 3:**
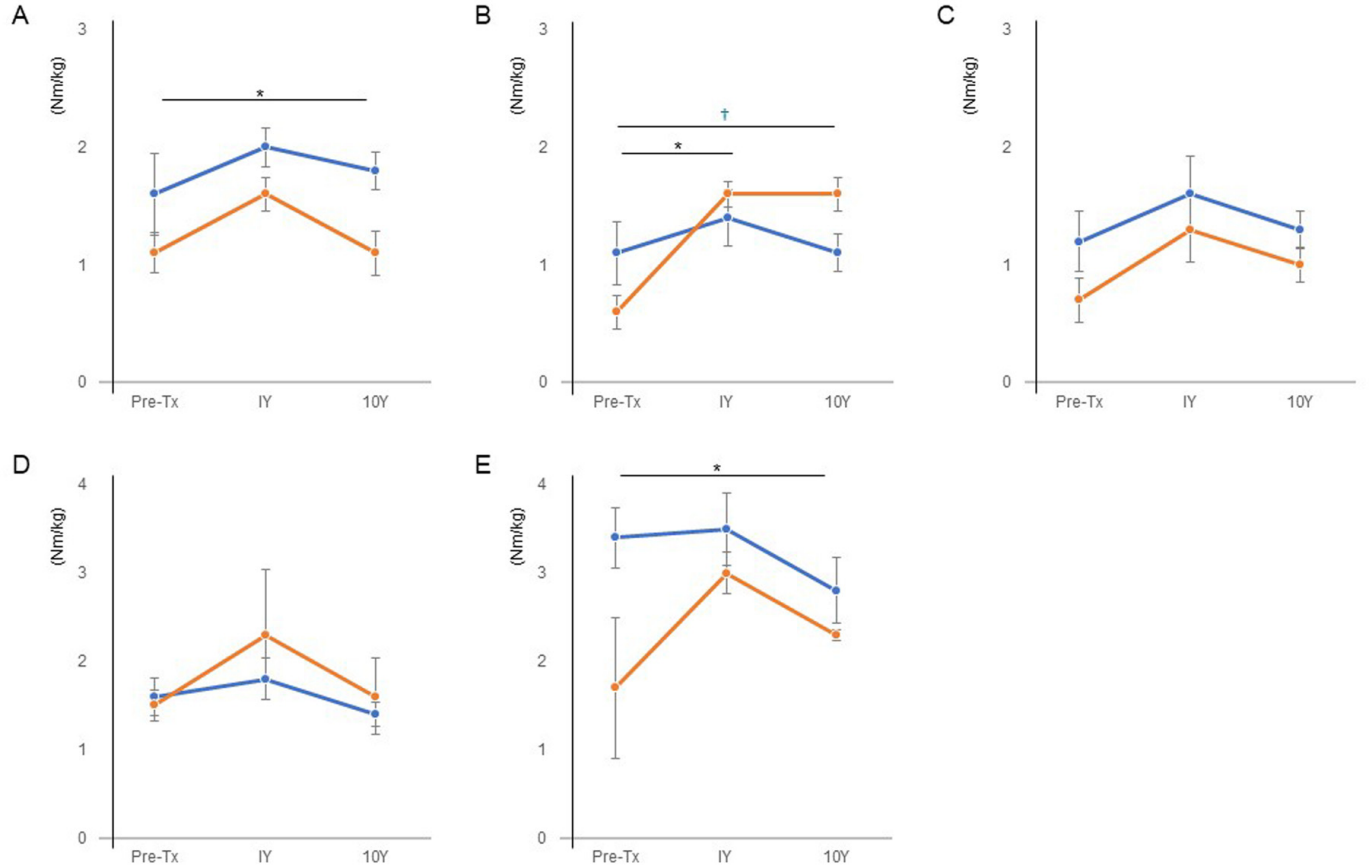
Transition of hip and knee muscle strength. (A) Hip flexor muscle strength. (B) Hip extensor muscle strength. (C) Hip abductor muscle strength. (D) Knee flexor muscle strength. (E) Knee extensor muscle strength (Nm/kg). The blue line indicates the average of the stable group (n=5); the orange line indicates the average of the progressed group without total hip arthroplasty (n=3). *Significant difference between pretreatment and 1 year after treatment. ^†^Significant difference between pretreatment and 10 years after treatment. *P*<.05. Tx, treatment; Y, year.

In both the stable group and the progressed group without total hip arthroplasty, the hip and knee muscle strength improved 1 year after the treatment but decreased slightly thereafter (fig 3). There was a significant improvement in hip extensor strength (fig 3B) in both the stable group and the progressed group without total hip arthroplasty; however, the difference between the groups was not significant. Hip flexor (fig 3A) and knee extensor strength (fig 3E) were significantly different between the stable group and the progressed group without total hip arthroplasty.

### Short Form-36 subgroup score

**Fig 4:**
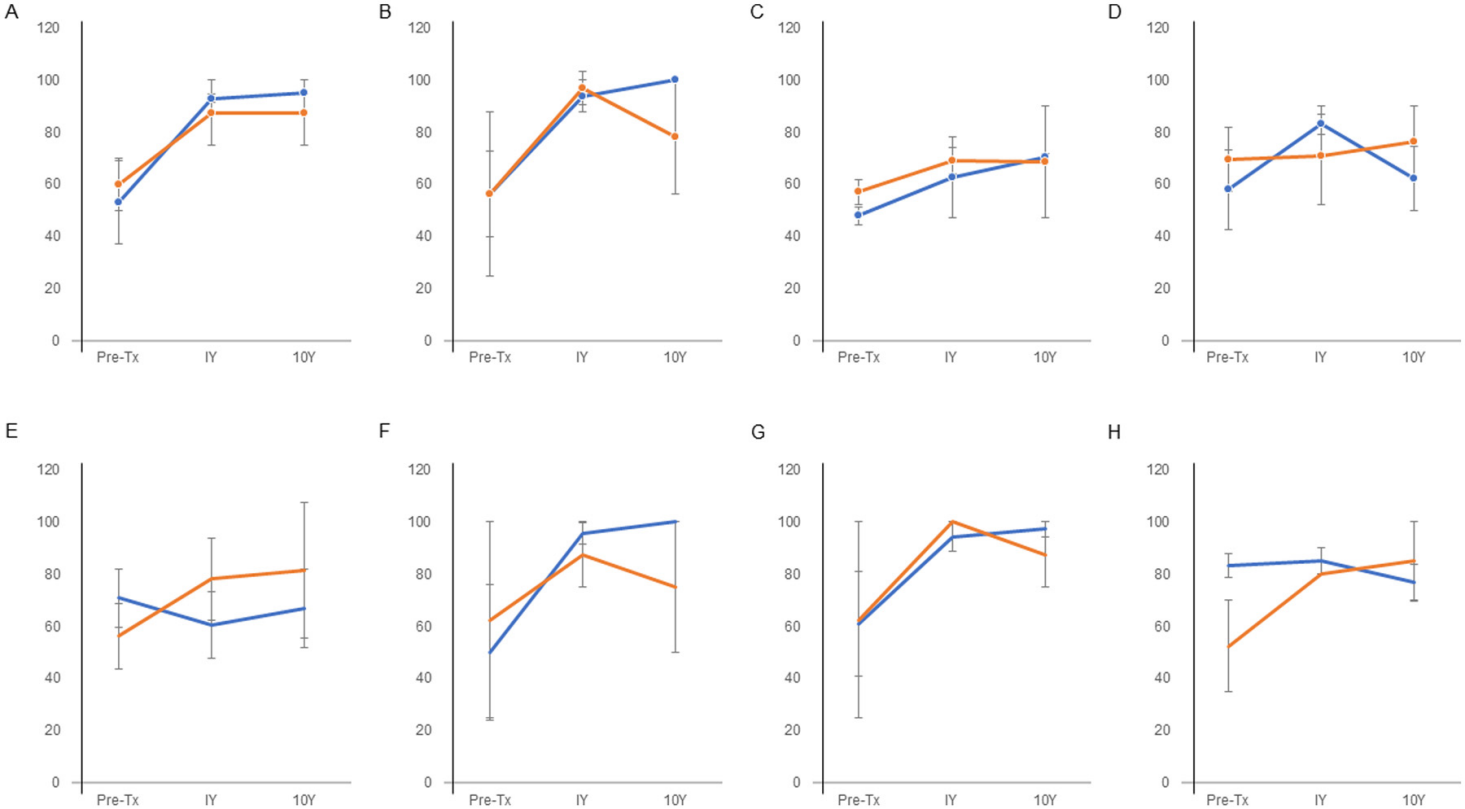
Transition of Short Form-36 subgroup scores: (A) physical function, (B) role limitations due to physical functioning, (C) bodily pain, (D) general health, (E) vitality, (F) social function, (G) role limitations due to emotional functioning, and (H) mental health. The blue line indicates the average of the stable group (n=3); the orange line indicates the average of the progressed group without total hip arthroplasty (n=2). Tx, treatment; Y, year.

There were improvements in physical functioning, role limitations due to physical functioning, bodily pain, general health, social functioning, and role functional subgroup scores in both the stable group (n=3) and the progressed group without total hip arthroplasty (n=2). However, statistical analysis was not performed because of the small number of cases (fig 4).

### Adverse events

There were no serious adverse events during the 10-year follow-up period.

## Discussion

In the current study, radiographic, clinical, physical function, and quality of life assessments were performed to evaluate the long-term effect of rehabilitation after mesenchymal stromal cell transplantation. Five of 10 hips (50%) did not progress to femoral head collapse; however, 5 (50%) did progress, and 2 hips (20%) in the progressed group required total hip arthroplasty. Compared with the findings of the 2-year follow-up study, 3 hips had progressed to collapse, and 2 hips required total hip arthroplasty.^10^ Considering the radiographic stages and necrotic areas, the results of the current study indicate the usefulness of our study procedure. Initially, all hips were at the postcollapse stage and had large necrotic areas. Without treatment, 96% of hips at the postcollapse stage and 84% of hips with large necrotic areas advanced to collapse.^22^ Even when bone marrow cells are transplanted, 57% of patients need total hip arthroplasty at this stage.^5^ Compared with the results of natural course and the outcomes of cell transplantation treatment, this study procedure was able to achieve successful outcomes.

The body mass index at 1 year after treatment was higher in the progressed group (23.7±0.6 kg/m^2^) than in the stable group (20.6±1.0 kg/m^2^). Moreover, clinical score, range of motion, muscle strength, and quality of life improved in both the stable group and the progressed group without total hip arthroplasty 10 years after treatment. The radiographic stage of the progressed group at pretreatment was more advanced than that of the stable group. Studies on the natural history of osteonecrosis of the femoral head suggest that the necrotic area is a risk factor for collapse; however, the radiographic stage is not a risk factor.^19^^,^^22^, ^23^, ^24^, ^25^ Although the radiographic stage alone is not a risk factor for collapse, when combined with a broad necrotic lesion, the difference in stage becomes a risk factor for collapse.^25^ In the current study, the necrotic area was broad in both the stable group (1 hip was type C1, 4 hips were type C2) and the progressed group (5 hips were type C2). Combined with the risk of collapse associated with a broad necrotic area, the stage might become a risk factor for collapse. Moreover, it is possible that some demographic factors combined with a broad necrotic area increase the risk of collapse. Studies of the natural history suggest that the demographic data of patients with osteonecrosis of the femoral head, such as sex, age, body mass index, steroid use, and smoking, are not independently associated with collapse.^24^^,^^25^ In the current study, body mass index was higher in the progressed group, especially in total hip arthroplasty cases (patients 3 and 7), than in the stable group (table 1). In the current rehabilitation program, one-third weight bearing was allowed 6 weeks after treatment followed by one-half weight bearing, two-thirds weight bearing, and full weight bearing at 2-week intervals in all patients. All patients were allowed to resume sports and work 6 months after the treatment. Although the level of weight bearing remains controversial,^26^ body weight affects weight bearing and social activity. The level and timing of weight bearing and social activity should be planned according to the patient's body composition. Lifestyle medicine has become increasingly important.^27^ Previously, lifestyle medicine was emphasized to control hypertension and diabetes,^27^ although recent research suggests that lifestyle rehabilitation is becoming more important to control locomotive disorders.^28^^,^^29^ Systematic lifestyle rehabilitation is needed in addition to exercise and cell transplantation.^29^

The findings of the previous 2-year follow-up study suggested that the rehabilitation program can feasibly improve physical function after cell transplantation for osteonecrosis of the femoral head.^12^ Notably, the clinical score, range of motion, and muscle strength were maintained 10 years after treatment, not only in the stable group, but also in the progressed group, without total hip arthroplasty. Functional exercise is effective in preventing inactivity and promoting early recovery from osteonecrosis of the femoral head.^30^ However, there are only a few reports about rehabilitation programs for osteonecrosis of the femoral head, especially regarding rehabilitation after cell transplantation.^8^^,^^12^ These previous studies focused on the timing and level of weight bearing^26^; however, few studies have evaluated programs that include range of motion, muscle strength, and aerobic exercises. Moreover, only a few reports described physical function outcomes such as range of motion, muscle strength, and quality of life after cell transplantation. Although previous cell transplantation studies without rehabilitation lack precise information about physical assessments,^8^^,^^9^ comparatively good results were achieved in the current study, reinforcing the findings of the previous feasibility study^12^ and indicating the usefulness of the examined treatment procedure for improving and maintaining clinical and hip function.

Recent research has provided new insights into regenerative rehabilitation to promote regeneration after cell transplantation.^31^ Locomotive training promoted the effect of neural cell transplantation for recovery and neurite extension in a rat brain injury model.^32^ Exercise promoted neurite extension from grafted dopaminergic neurons in a rat model of Parkinson disease.^33^ Treadmill exercise and mesenchymal stromal cell transplantation enhanced cartilage repair in a rat osteochondral defect model.^34^ Extrinsic mechanical cues are transmitted to cells and regulate gene expression via cytoskeletal structures. During development, shear, tensile, and compressive mechanical pressure play a role in morphogenesis, stimulating tissue-specific stem cells.^17^ Moreover, during exercise, skeletal muscles release many cytokines such as insulin-like growth factor, brain-derived neurotrophic factor, interleukin-6, and myostatin.^35^, ^36^, ^37^ Mechanical stimuli and cytokines may enhance the function of stem cells for tissue regeneration. In the current study, although we did not demonstrate such a synergistic effect of rehabilitation combined with cell transplantation, the findings suggest that regenerative rehabilitation is possible.

### Study limitations

The current study has several major limitations. This was a small-scale, single-group, pre-post study without a control group. There is potential bias because of the small sample size. The current study was a secondary study of the original^10^ and 10-year follow-up studies.^11^ The measured parameters were not planned only for the current study. A clinical trial with an adequate study design regarding outcome, number of participants, and risk analysis is needed to demonstrate the precise effect of rehabilitation after cell transplantation.^18^ The current study was generalized because it was a new clinical trial.

## Conclusions

The current study reports the effects of a rehabilitation program after mesenchymal stromal cell transplantation for osteonecrosis of the femoral head 10 years after treatment and rehabilitation. The 12-week rehabilitation program and annual follow-up after mesenchymal stromal cell transplantation for osteonecrosis of the femoral head was associated with reducing pain reduction, maintaining hip muscle strength, widening range of motion, and improving quality of life. Long-term lifestyle rehabilitation programs such as those involving weight control and activity level according to the individual's lifestyle and body composition are needed. Although this study is limited in showing the precise effect of rehabilitation after cell transplantation, the results of the current study may promote the science of regenerative medicine and rehabilitation medicine.

## Suppliers

a. Osferion; Olympus Terumo Biomaterials Co. b. Handheld dynamometer; Nihon Medix Co Ltd. c. Iso Force GT-330; OG Giken Co Ltd, Okayama, Japan. d. JMP IN, version 15; SAS Institute Inc.

## Appendix Supplementary materials

**Supplementary Fig S1** Transition of radiographic stages during the 10 years after treatment. ●stage 3A ▲stage 3B ■stage 4 □total hip arthroplasty (THA). Image, image 1
