# Supplementary figures and images for: Effect of a Rehabilitation Program After Mesenchymal Stromal Cell Transplantation for Advanced Osteonecrosis of the Femoral Head: A 10-Year Follow-Up Study
Source: Arch Rehabil Res Clin Transl. 2022 Jan 13;4(1):100179. doi: 10.1016/j.arrct.2022.100179 (PMC8904865; doi:10.1016/j.arrct.2022.100179)

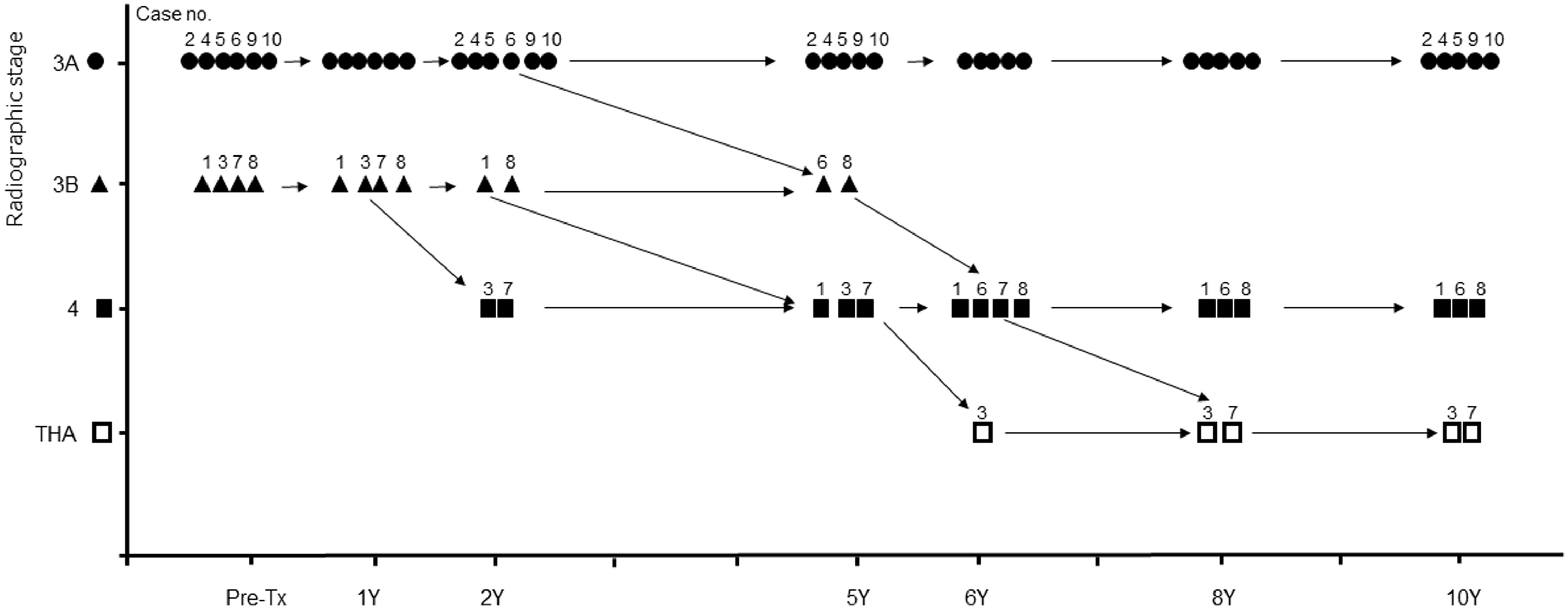

Supplement: Supplementary file 1 — Supplementary Fig S1 Transition of radiographic stages during the 10 years after treatment. ●stage 3A ▲stage 3B ■stage 4 □total hip arthroplasty (THA). [file mmc1.jpg]
